# *Depdc5* deficiency exacerbates alcohol-induced hepatic steatosis via suppression of PPARα pathway

**DOI:** 10.1038/s41419-021-03980-6

**Published:** 2021-07-15

**Authors:** Lin Xu, Xinge Zhang, Yue Xin, Jie Ma, Chenyan Yang, Xi Zhang, Guoqing Hou, Xiaocheng Charlie Dong, Zhaoli Sun, Xiwen Xiong, Xuan Cao

**Affiliations:** 1 Department of Medical Genetics, School of Basic Medicine, Tongji Medical College, Huazhong University of Science and Technology, Wuhan, Hubei 430030 PR China; 2 School of Forensic Medicine, Xinxiang Medical University, Xinxiang, Henan 453003 PR China; 3 Xinxiang Key Laboratory of Metabolism and Integrative Physiology, Xinxiang Medical University, Xinxiang, Henan 453003 PR China; 4 Department of Human Anatomy & Histoembryology, School of Basic Medical Sciences, Xinxiang Medical University, Xinxiang, Henan 453003 PR China; 5 Department of Biochemistry and Molecular Biology, Indiana University School of Medicine, Indianapolis, IN 46202 USA; 6 Department of Surgery, Johns Hopkins University School of Medicine, Baltimore, MD 21205 USA

**Keywords:** Alcoholic liver disease, Experimental models of disease

## Abstract

Alcohol-related liver disease (ALD), a condition caused by alcohol overconsumption, occurs in three stages of liver injury including steatosis, hepatitis, and cirrhosis. DEP domain-containing protein 5 (DEPDC5), a component of GAP activities towards Rags 1 (GATOR1) complex, is a repressor of amino acid-sensing branch of the mammalian target of rapamycin complex 1 (mTORC1) pathway. In the current study, we found that aberrant activation of mTORC1 was likely attributed to the reduction of DEPDC5 in the livers of ethanol-fed mice or ALD patients. To further define the in vivo role of DEPDC5 in ALD development, we generated *Depdc5* hepatocyte-specific knockout mouse model (Depdc5-LKO) in which mTORC1 pathway was constitutively activated through loss of the inhibitory effect of GATOR1. Hepatic *Depdc5* ablation leads to mild hepatomegaly and liver injury and protects against diet-induced liver steatosis. In contrast, ethanol-fed Depdc5-LKO mice developed severe hepatic steatosis and inflammation. Pharmacological intervention with Torin 1 suppressed mTORC1 activity and remarkably ameliorated ethanol-induced hepatic steatosis and inflammation in both control and Depdc5-LKO mice. The pathological effect of sustained mTORC1 activity in ALD may be attributed to the suppression of peroxisome proliferator activated receptor α (PPARα), the master regulator of fatty acid oxidation in hepatocytes, because fenofibrate (PPARα agonist) treatment reverses ethanol-induced liver steatosis and inflammation in Depdc5-LKO mice. These findings provide novel insights into the in vivo role of hepatic DEPDC5 in the development of ALD.

## Introduction

Alcohol-related liver disease (ALD) encompasses a broad spectrum of progressive liver pathologies that range from simple steatosis to severe forms of liver injury, such as steatohepatitis, liver fibrosis, cirrhosis, and even hepatocellular carcinoma (HCC) [1]. Since no effective therapies for end-stage ALD are currently available, there is a clinical need for better understanding of the pathogenesis of ALD and identification of therapeutic targets.

Mammalian target of rapamycin complex 1 (mTORC1) is a highly conserved protein kinase complex that regulates cell growth and metabolism in response to growth factor, nutrient abundance and cellular stress [2]. The tuberous sclerosis complex (TSC), consisting of TSC1, TSC2, and TBC1D7, is a major mediator of growth factor and cellular stress signaling to mTORC1. TSC acts as a GTPase-activating protein (GAP) for Rheb and serves as a negative regulator of mTORC1 signaling [3]. The GAP activities towards Rags 1 complex (GATOR1), which consists of DEP domain-containing protein 5 (DEPDC5), NPR2-like GATOR1 complex subunit (NPRL2), and NPR3-like GATOR1 complex subunit (NPRL3), is a pivotal negative regulator of mTORC1 activation in response to amino acids. GATOR1 prevents translocation of mTORC1 to its active site at the lysosome membrane during amino acid insufficiency by acting as a GAP to keep Rag A/B in the inactive GDP state [4]. As a component of GATOR1 complex, DEPDC5 functions as a GAP for Rag A/B [4]. *DEPDC5* was identified as a gene responsible for familial focal epilepsy [5, 6]. Germline homozygous knockout *Depdc5* rat and mouse models are embryonic lethal due to mTORC1 hyperactivation [7, 8]. Importantly, genetic variations in the *DEPDC5* locus were associated with hepatitis C virus (HCV)-induced fibrosis or HCC progression in humans [9, 10].

As one of the essential metabolic organs, the liver requires proper regulation of mTORC1 activity for maintaining homeostasis and preventing pathologies [11]. Disrupting mTORC1 through liver-specific deletion of regulatory-associated protein of mTOR (Raptor) in mice inhibits the sterol regulatory element-binding protein1 (SREBP1) target gene expression and suppresses liver fat accumulation in response to a high-fat and high-cholesterol diet feeding [12]. Surprisingly, activating mTORC1 through liver-specific deletion of *Tsc1* does not induce fat accumulation in the liver and even protects the liver against high-fat diet (HFD)-induced hepatic steatosis [13, 14]. A recent study has demonstrated that liver-specific *Depdc5* knockouts exhibit many phenotypes similar to *Tsc1* knockout mice, such as elevated inflammation and resistance to HFD-induced steatosis [15]. Although great progress has been made in unraveling how mTORC1 regulates the pathophysiology of nonalcoholic fatty liver disease (NAFLD), relatively little is known about the role of mTORC1 pathway in ALD. In this study, we aimed to examine the in vivo role of aberrant mTORC1 activation caused by *Depdc5* deletion in regulating pathogenesis of ALD.

## Materials and methods

### Human liver tissue samples

Control and alcoholic hepatitis (AH) human liver samples were obtained under the institutional review board protocol approved by Johns Hopkins University School of Medicine (Table S1).

### Animal studies

The study was approved by the Institutional Animal Care and Use Committee of Xinxiang Medical University, China. All animal procedures were performed in accordance with “Guide for the Care and Use of Laboratory Animals” published by the National Institutes of Health. Euthanasia was performed using compressed carbon dioxide (CO_2_) from gas cylinders or cervical dislocation under anesthesia. Group allocation for the experiments was randomized and not blinded. Sample analyses were not blinded.

*Depdc5* floxed mice (*Depdc5*^*flox/flox*^), originated from the ES clone (Clone No. HEPD0734_3_G10), were provided by the CAM-SU Genomic Resource Center, Soochow University (Suzhou, China), one of the mutant ES cell repositories for Asian-Pacific research community in International Mouse Phenotyping Consortium (IMPC). The hepatocyte-specific *Depdc5* knockout mice were generated by crossing *Depdc5*^*flox/flox*^ mice with *Albumin (Alb)-Cre* mice.

Both 2–3-month-old male and female mice were used in generating mouse models of ALD. We generated two mouse ALD models in the current study: (1) Chronic-plus-binge ethanol feeding model (Gao-Binge model). The Gao-Binge ethanol feeding protocol as described previously [16]. (2) Lieber-DeCarli liquid diet feeding model (LD 5W model). Mice were fed a Lieber-DeCarli liquid diet containing 5% ethanol (v/v) or an isocaloric control diet for 5 weeks. Then, mice were euthanized without ethanol binge and, blood and liver tissues were collected.

For Torin 1 treatment, LoxP and Depdc5-LKO mice received daily i.p. injections of 20 mg/kg Torin 1 (dissolved in 20% *N*-methyl-2-pyrrolidone/40% PEG400/40% H_2_O) or vehicle from the 6th day to the 10th day of ethanol diet feeding. On the 11th day morning, mice were injected with Torin 1 immediately after ethanol binge and were sacrificed 9 h later. For fenofibrate treatment, 3-month-old LoxP and LKO mice were fed 5% ethanol diet containing 0.02% fenofibrate for 10 days and then binged with ethanol at 8:00 am of the 11th day, and were sacrificed 9 h later. For adenovirus-mediated liver-specific gene knockdown, Ad-shGFP vs Ad-shRaptor adenoviruses were delivered via tail vein injection (1 × 10^9^ pfu/mouse) into 3-month-old LoxP and LKO mice on the 1st day of ethanol diet feeding. Mice were binged with ethanol on the 11th day morning and were sacrificed 9 h later.

### Adenovirus preparation

Ad-shRaptor and control Ad-shGFP vectors were generated using the pAdBLOCK-iT system (Invitrogen, USA). Adenoviruses were amplified in HEK293A cells and purified by CsCl gradient centrifugation. The adenoviruses were titered using an Adeno-XTM Rapid Titer kit (Takara Bio, China) according to the manufacturer’s manual.

### Western blot analysis

Protein extracts from hepatocytes or liver tissues were made in RIPA buffer supplemented with 1 mM phenylmethylsulfonyl fluoride (PMSF) and Roche cOmplete protease inhibitor cocktail (Roche, Germany). Protein extracts were separated on an SDS-PAGE gel, transferred to nitrocellulose membranes, and blotted with the indicated primary antibodies at 4 °C for overnight. Horseradish peroxidase (HRP)-conjugated secondary antibodies were given for 1 h. The immune complexes were detected using the ECL detection reagents (Beyotime, China). Band densitometries were obtained with ImageJ software. Detailed information of the primary antibodies used in this study is listed in Table S2.

### Real-time RT-PCR analysis

Total RNAs were extracted from liver tissues using TRIzol reagent (Takara Bio, China) and converted into cDNAs using a cDNA synthesis kit (Vazyme, China). Real-time PCR analysis was performed using SYBR Green Master Mix (Vazyme, China) in ABI StepOnePlus Real-Time PCR system. Sequence information of the primers used in this study is listed in Table S3.

### RNA sequencing

The total liver RNAs from alcohol-fed LoxP and Depdc5-LKO mice (3 biological replicates) were used for RNA-seq. The RNA-seq libraries were prepared by the Beijing Genomics Institute and sequenced using BGISEQ-500 platform. Genes that significantly and differentially expressed between LoxP and LKO mice were selected based on a fold change >2.0 and a *p*-value < 0.05, and subsequently analyzed by Kyoto Encyclopedia of Genes and Genomes (KEGG) pathway enrichment analysis. The original RNA-seq data have been submitted to the database of NCBI Sequence Read Archive under the accession number SUB9331871.

### Histology

Liver tissues were fixed in 4% paraformaldehyde (PFA), embedded in paraffin and sectioned at a thickness of 5 µm. Sections were stained with hematoxylin-eosin (H&E) according to standard procedures. Immunohistochemistry (IHC) analysis for detecting oxidative stress (anit-4-HNE) and inflammation (anit-F4/80 and anti-MPO) was performed using IHC detection kit (ZSGB Bio, China) following manufacture’s manual. For Oil Red O staining, 4% PFA-fixed liver tissues were embedded in OCT, sectioned at 10 µm, and then stained with 0.5% Oil Red O according to standard procedures.

### Transmission electron microscopy

Mouse liver tissues (~1 mm^3^) were fixed with 2% glutaraldehyde for 2 h, followed by 1% osmium tetroxide for 2 h. After dehydration and embedding in resin, ultra-thin sections (80 nm) were stained with uranyl acetate and lead citrate and analyzed in HT7800 transmission electron microscope (TEM) (Hitachi, Japan).

### Biochemical analysis

Hepatic lipids were extracted with chloroform/methanol (2:1), as described previously [17]. Liver triglyceride (TG) levels were determined using triglyceride quantification kit (Sigma, USA) and normalized with liver tissue weights. Serum alanine aminotransferase (ALT) levels were analyzed using ALT assay kit (Nanjing Jiancheng, China)

### Statistical analysis

All data are presented as mean ± SD. Using GraphPad Prism 8 software, data were analyzed by one-way analysis of variance between multiple groups, when appropriate, and by a two-tailed unpaired Student’s *t*-test between two groups. *p* < 0.05 was considered as significant.

## Results

### Deletion of hepatic *Depdc5* activates mTORC1 signaling and results in mild liver injury

**Fig. 1:**
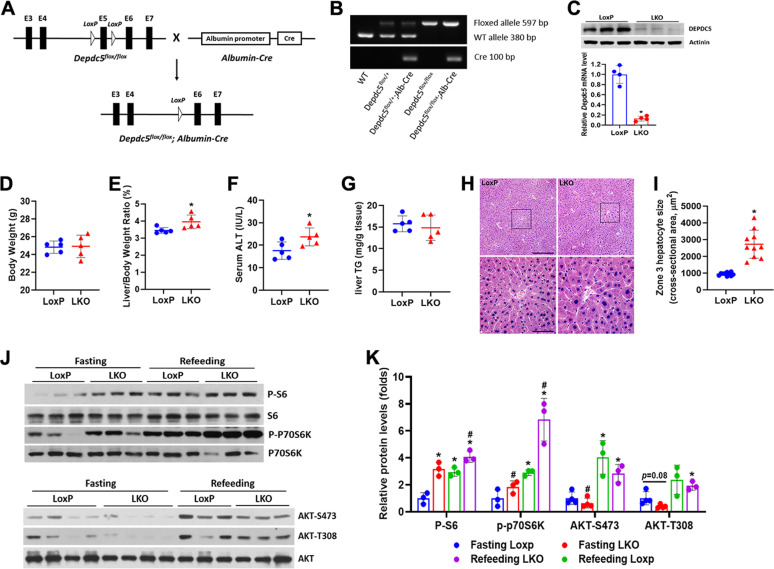
Hepatocyte-specific *Depdc5* deletion induces upregulation of mTORC1 activity and mild liver injury. Two-month-old LoxP mice and Depdc5-LKO mice were subjected to the following analyses. **A**, **B** The strategy for creating *Depdc5* hepatocyte-specific knockout mice (**A**) used in the current study and a representative gel electrophoresis image for genotyping (**B**). **C** Western blot and qPCR analyses of DEPDC5 in the livers of control LoxP and LKO mice (*n* = 4/group). **D**, **E** Body weights (**D**) and liver-weight-to-body-weight ratios (**E**) of LoxP and LKO mice (*n* = 5/group). **F**, **G** Serum ALT (**F**) and hepatic TG (**G**) measurements of LoxP and LKO mice (*n* = 5/group). **H** Representative H&E images of liver sections from LoxP and LKO mice; the rectangular boxed areas in upper panels (100×) are magnified as shown in lower panels (400×). **I** Calculation of the cross-sectional area of zone 3 hepatocytes by ImageJ. **J** Western blot analysis of liver lysates from overnight fasting or 4 h refeeding LoxP and LKO mice are shown for the indicated phospho-(P) and total proteins. **K** Quantification of band intensities of phosphorylated proteins normalized to total proteins in (**J**). Data are presented as mean ± SD. *p < 0.05. In (**K**), *p < 0.05 vs Fasting LoxP; ^#^*p* < 0.05 vs Refeeding LoxP. Scale bars: (**H**) upper, 200 µm; (**H**) lower, 50 µm.

*Depdc5* floxed mice were crossed with *Albumin-Cre* mice to generate hepatocyte-specific *Depdc5* knockout mice (referred to as Depdc5-LKO mice, genotype: *Depdc5*^*flox/flox*^, *Cre*) and control mice (referred to as LoxP, genotype: *Depdc5*^*flox/flox*^) (Fig. 1A, B). Deletion of *Depdc5* in the liver of LKO mice was confirmed at both mRNA and protein levels (Fig. 1C). Liver-weight-to-body-weight ratios were significantly higher in Depdc5-LKO mice compared to LoxP mice (Fig. 1D, E). H&E staining of liver sections revealed that Depdc5-LKO mice had specific enlargement of pericentral zone 3 hepatocytes (Fig. 1H, I). Depdc5-LKO mice had a significant elevation of serum ALT level, indicating a mild liver damage caused by *Depdc5* ablation (Fig. 1F). Unexpectedly, the enlarged liver observed in Depdc5-LKO mice cannot be associated with hepatic steatosis, because Depdc5-LKO mice exhibited even slightly reduced liver triglyceride levels compared to LoxP mice (Fig. 1G). To further evaluate the effect of *Depdc5* deficiency on hepatic lipid metabolism, we challenged Depdc5-LKO mice with a HFD (60% kcal fat). Interestingly, Depdc5-LKO mice exhibited protection from HFD-induced hepatic steatosis (Fig. S1A–D). Hepatic deletion of *Depdc5* led to sustained activation of mTORC1 signaling even under fasting state, as shown by increased expression of P-S6K1 (Thr389) and P-S6 (Ser235/236) (Fig. 1J, K). Since constitutive activation of mTORC1 is known to suppress the activity of AKT secondary to feedback inhibition of activated S6K onto insulin receptor substrate 1 (IRS1), we observed that hepatic AKT phosphorylation at Ser473 and Thr308 was suppressed in both fasting and refeeding states in Depdc5-LKO mice (Fig. 1J, K).

### Hepatic DEPDC5 is downregulated by chronic ethanol consumption

**Fig. 2:**
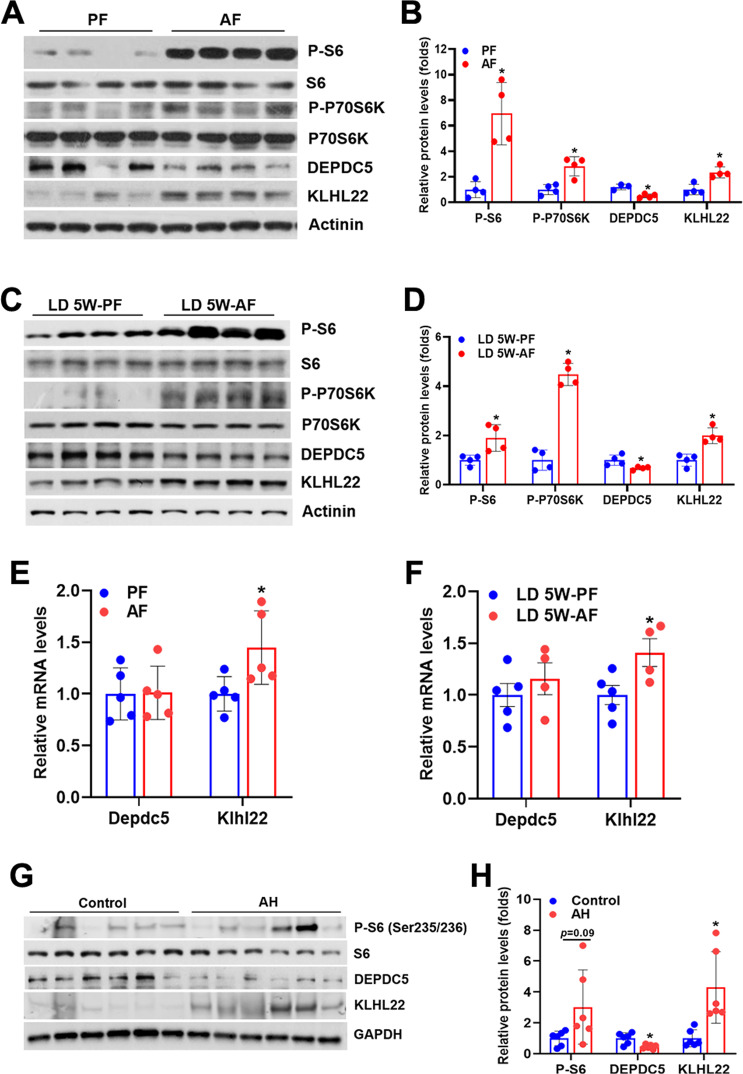
Chronic alcohol consumption reduces DEPDC5 protein while activates mTORC1 signaling in the liver. Two–three-month-old LoxP and Depdc5-LKO mice were subjected to chronic-plus-binge ethanol feeding (Gao-Binge model) or Lieber-DeCarli liquid diet containing 5% ethanol feeding for 5 weeks (LD 5W model). **A**, **B** Western blot (**A**) and quantification (**B**) analyses of hepatic mTORC1 signaling, DEPDC5, and KLHL22 in mice treated with Gao-Binge ethanol feeding. **C**, **D** Western blot (**C**) and quantification (**D**) analyses of hepatic mTORC1 signaling, DEPDC5, and KLHL22 in mice treated with LD 5W ethanol feeding. **E**, **F** qPCR analysis of mRNA expression of *Depdc5* and *Klhl22* genes in the livers of mice treated with Gao-Binge ethanol feeding (**E**) or LD 5W ethanol feeding (**F**) and their corresponding pair-fed controls (*n* = 4–5/group). **G**, **H** Western blot and quantification analyses of mTORC1 signaling, DEPDC5, and KLHL22 in the liver samples of normal controls and AH patients. Data are presented as mean ± SD. *p < 0.05.

To test whether DEPDC5 contributes to alcohol-induced mTORC1 activation in the liver, we then determined hepatic DEPDC5 expression in two well-established mouse models of ALD, the chronic-plus-binge ethanol feeding model (Gao-Binge model) [16] and the Lieber-DeCarli ethanol diet feeding model (mice fed a 5% ethanol diet for 5 weeks, referred to as LD 5W model). As expected, ethanol administration by either approach led to a robust activation of mTORC1 signaling in the liver (Fig. 2A–D). In both ALD models, hepatic DEPDC5 protein levels were remarkably reduced in ethanol-treated mice compared to their isocaloric pair-fed controls (Fig. 2A–D). Interestingly, the reduced DEPDC5 protein levels were not due to the suppression of *Depdc5* transcription as evidenced by unchanged *Depdc5* mRNA levels in the liver tissues of mice treated with ethanol by either approach (Fig. 2E, F). A recent study has revealed that kelch-like family member 22 (KLHL22), an adaptor protein of the CUL3–RBX1–KLHL22 E3 ligase complex, regulates the ubiquitination and degradation of DEPDC5 in response to amino acid availability [18]. We hypothesized that the decrease of hepatic DEPDC5 protein in ALD mice was due to enhanced degradation mediated by KLHL22. Indeed, a robust increase of hepatic KLHL22 expression was observed in both mouse models of ALD (Fig. 2A–F). To gain clinical evidence for the relationship among mTORC1 signaling, DEPDC5, and KLHL22, we analyzed their protein levels in the liver samples from healthy controls and patients with AH. As expected, we found that phospho-S6 (S235/236) and KLHL22 protein levels were markedly elevated, whereas DEPDC5 protein levels were significantly reduced in the liver tissues from AH patients compared to those from controls (Fig. 2G, H).

### Hepatic loss of *Depdc5* exacerbates alcohol-induced steatosis

**Fig. 3:**
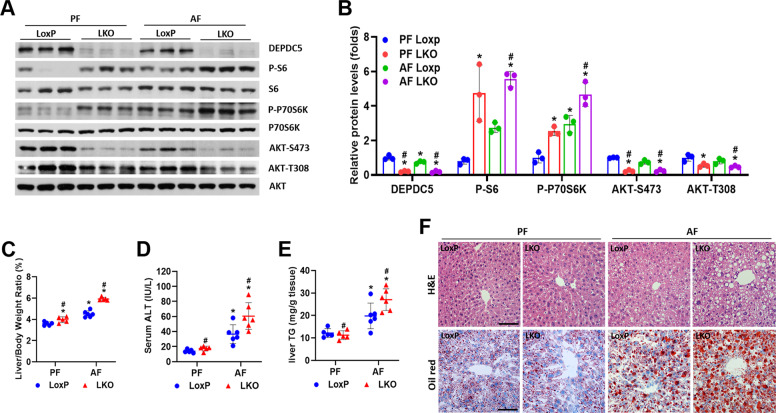
Hepatic deletion of *Depdc5* provokes ethanol-induced liver steatosis. Two–three-month-old LoxP and Depdc5-LKO mice were subjected to Gao-Binge ethanol feeding. **A**, **B** Liver lysates were subjected to western blot (**A**) and quantification (**B**) analyses to examine mTORC1 signaling and AKT phosphorylation. **C**–**E** Liver-weight-to-body-weight ratios (**C**), serum ALT levels (**D**), and liver TG levels (**E**) were determined (*n* = 5–6/group). **F** Liver sections were analyzed through H&E and Oil Red O staining (200×). Data are presented as mean ± SD. *p < 0.05 vs PF LoxP; ^#^*p* < 0.05 vs AF LoxP. Scale bars, 100 µm.

Chronic activation of mTORC1 signaling in hepatocytes protects against diet-induced hepatic steatosis [13–15]. However, the consequences of mTORC1 signaling hyperactivation in the development of ALD are still unknown. To investigate the role of hepatic *Depdc5* deletion mediated hyperactivation of mTORC1 in ALD pathogenesis, Depdc5-LKO, and control LoxP mice were subjected to Gao-Binge ethanol feeding. *Depdc5* ablation and ethanol exposure synergistically activated mTORC1 signaling and further inhibited AKT activity through feedback loop in the liver (Fig. 3A, B). Moreover, ethanol-fed Depdc5-LKO mice exhibited severely impaired liver autophagy, as demonstrated by dramatic accumulation of p62 and reduced LC3-II protein (Fig. S2A). In addition, the expression of ER stress markers (CHOP, BiP, ATF4, XBP1s, and ATF6) was significantly elevated in the liver tissues of ethanol-fed Depdc5-LKO mice (Fig. S2B). Gao-Binge ethanol-fed Depdc5-LKO mice exhibited the most dramatic increase of liver-weight-to-body-weight ratios and had the highest serum ALT levels among the four groups of mice we studied (Fig. 3C, D). Surprisingly, in contrast to what we observed in HFD feeding experiment, Gao-Binge ethanol feeding remarkably elevated hepatic triglyceride levels in Depdc5-LKO mice compared to LoxP mice (Fig. 3E, F). We next examined whether hepatic *Depdc5* deficiency contributes to the development of alcohol-induced steatosis in another ALD mouse model, LD 5W model. Notably, we also observed increased liver-weight-to-body-weight ratios, liver triglyceride contents, and serum ALT levels in Depdc5-LKO mice compared to LoxP mice, indicating *Depdc5* deletion-mediated mTORC1 activation worsens the hepatic steatosis induced by ethanol (Fig. S3A–D).

### Deletion of hepatic *Depdc5* aggravates alcohol-induced oxidative stress and inflammation

**Fig. 4:**
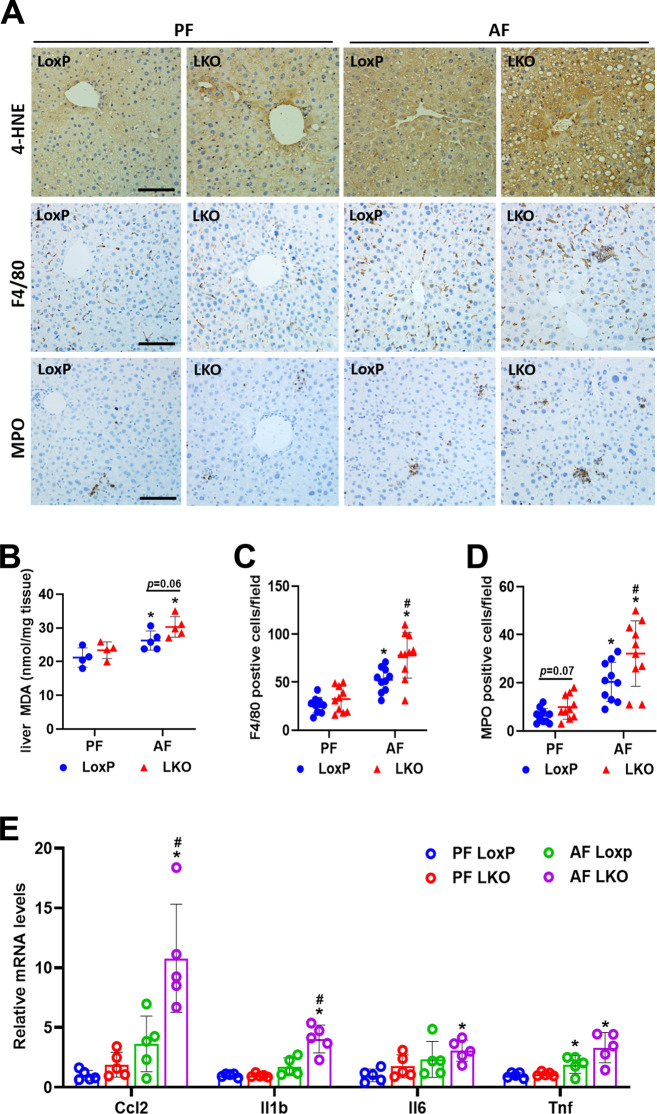
Hepatic ablation of *Depdc5* aggravates ethanol-induced oxidative stress and inflammation in the liver. Two–three-month-old LoxP and Depdc5-LKO mice were subjected to Gao-Binge ethanol feeding. **A** IHC analysis of 4-HNE, F4/80, and MPO in liver sections (200×, *n* = 3–4/group). **B** Hepatic MDA measurements (*n* = 4–5/group). **C**, **D** Quantification of F4/80-positive cells (**C**) and MPO-positive cells (**D**) shown in (**A**). **E** qPCR analysis of hepatic mRNA expression of inflammation-related genes (*n* = 5/group). Data are presented as mean ± SD. *p < 0.05 vs PF LoxP; ^#^*p* < 0.05 vs AF LoxP. Scale bars, 100 µm.

In addition to inducing lipid dysregulation, mTORC1 hyperactivation also results in other liver abnormalities, e.g., oxidative stress and inflammation [15, 19]. Indeed, ethanol-fed Depdc5-LKO mice had higher levels of oxidative stress in the liver, as indicated by an elevation of lipid peroxidation markers, 4-hydroxynonenal (4-HNE) and malondialdehyde (MDA) (Fig. 4A, B). Moreover, ethanol-fed Depdc5-LKO mice had significantly more macrophages (F4/80 positive) and neutrophils (MPO positive) in the liver than LoxP mice, indicating hyperactivation of mTORC1 signaling worsened ethanol-induced hepatic inflammation (Fig. 4A, C, D). Consistently, the mRNA expression of proinflammatory cytokines including *Il6*, *Il1b*, *Ccl2*, *Tnf* was also highly induced in the liver tissues of ethanol-fed Depdc5-LKO mice (Fig. 4E). To investigate whether hepatic deletion of *Depdc5* aggravates ethanol-induced liver fibrosis, we next investigate the expression of fibrogenic markers. Unexpectedly, no significant alterations in hepatic expression of fibrosis-related genes were noted in ethanol-fed LoxP and Depdc5-LKO mice (Fig. S4A, B). Sirius Red staining also revealed comparable deposition of collagen contents between both genotypes of mice fed an ethanol diet (Fig. S4C).

### Pharmacologic inhibition of mTOR rescues ethanol-induced hepatic steatosis and inflammation in Depdc5-LKO mice

To test whether the ethanol-induced liver pathologies in Depdc5-LKO mice were mainly due to mTORC1 activation, we took advantage of adenoviral shRNA expression system to create a relatively liver-specific knockdown of *Raptor*, which is the key scaffolding protein of the mTORC1 complex [2]. Ad-shRaptor markedly decreased hepatic RAPTOR protein levels and suppressed mTORC1 signaling activity (Fig. S5A). *Raptor* knockdown significantly reversed the ethanol-induced hepatic steatosis caused by *Depdc5* deletion (Fig. S5B, D, E). In addition, *Raptor* knockdown slightly ameliorated liver injury and inflammation in Depdc5-LKO mice as well (Fig. S5C, F–H). Surprisingly, LoxP mice that received Ad-shRaptor had elevated serum ALT levels and increased hepatic immune cells infiltration compared to LoxP mice that received Ad-shGFP (Fig. S5C, F–H), which is consistent with the previous study that hepatocyte-specific deletion of *Raptor* results in liver damage and a marked enhancement of hepatocarcinogenesis [20].

**Fig. 5:**
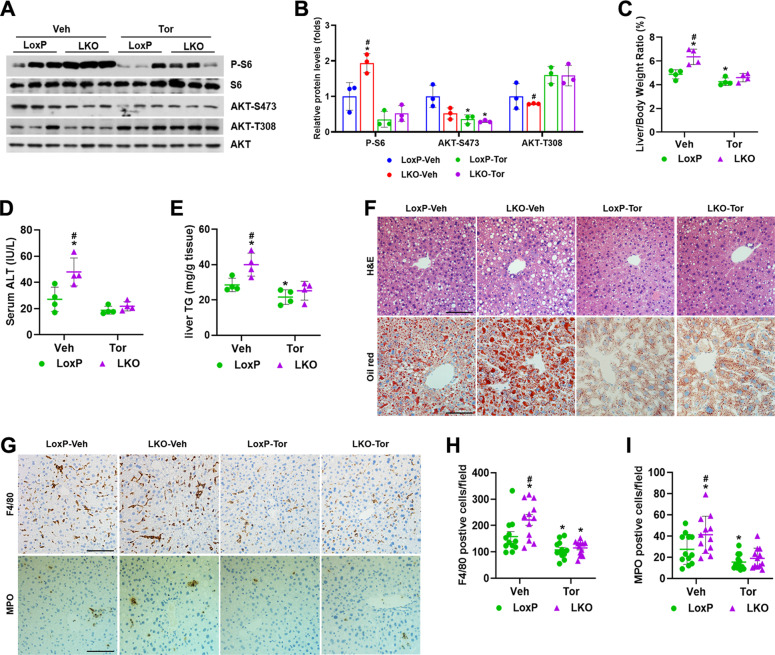
Torin 1 administration rescues Gao-Binge ethanol feeding-induced liver abnormalities in Depdc5-LKO mice. Three-month-old male LoxP and Depdc5-LKO mice were subjected to Gao-Binge ethanol feeding. **A**, **B** Liver lysates were subjected to western blot (**A**) and quantification (**B**) analyses to examine mTORC1 signaling and AKT phosphorylation. **C**–**E** Liver-weight-to-body-weight ratios (**C**), serum ALT levels (**D**) and liver TG levels (**E**) were determined (*n* = 4/group). **F** Liver sections were analyzed through H&E and Oil Red O staining (200×). **G** Hepatic macrophages and neutrophils were examined by IHC staining (200×) with anti-F4/80 and anti-MPO antibodies (*n* = 4/group). **H**, **I** Quantification of F4/80-positive cells (**H**) and MPO-positive cells (**I**) shown in (**G**). Data are presented as mean ± SD. *p < 0.05 vs LoxP-Veh; ^#^*p* < 0.05 vs LoxP-Tor. Scale bars, 100 µm.

Ad-shRaptor-mediated *Raptor* knockdown led to persistent inactivation of mTORC1 and exacerbated ethanol-induced liver injury. We took advantage of Torin 1, an ATP-competitive mTOR inhibitor that inhibits mTORC1 signaling [21], to modestly suppress mTORC1 activity in ethanol-fed Depdc5-LKO and LoxP mice. Torin 1 treatment remarkably blocked the phosphorylation of S6 and AKT (Ser473), whereas induced the phosphorylation of AKT (Thr308) due to inhibition of the mTORC1-dependent negative feedback loop (Fig. 5A, B). After Torin 1 administration, liver-weight-to-body-weight ratios, liver triglyceride levels and serum ALT levels showed dramatic recovery, indicating that sustained hepatic mTORC1 activation is indeed the major cause of hepatic steatosis observed in Depdc5-LKO mice (Fig. 5C–F). In addition, we also analyzed the effect of Torin 1 administration on hepatic inflammation. Analysis of F4/80 and myeloperoxidase (MPO) staining demonstrated that Torin 1 treatment markedly reduced the number of macrophages and neutrophils in the liver tissues of both genotypes of mice (Fig. 5G–I).

### Hepatic loss of *Depdc5* suppresses peroxisome proliferator activated receptor α (PPARα) activity and damages mitochondria

**Fig. 6:**
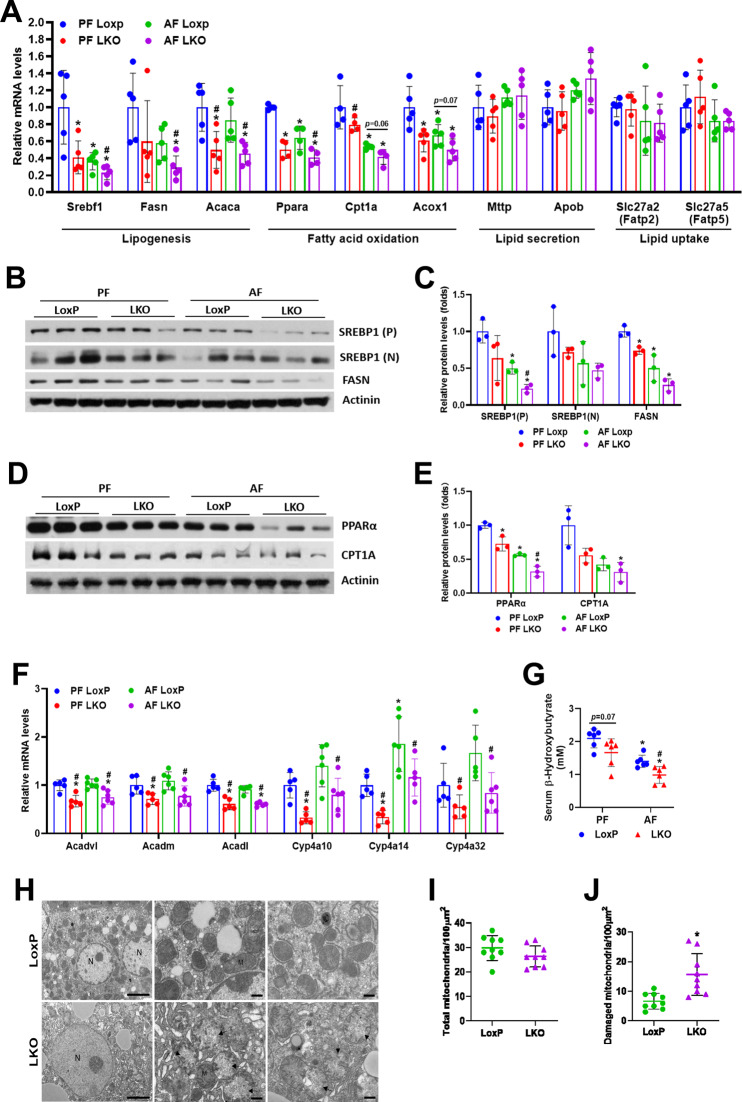
Hepatic ablation of *Depdc5* inhibits the expression of PPARα and its target genes and results in mitochondrial damage. Two–three-month-old LoxP and Depdc5-LKO mice were subjected to Gao-Binge ethanol feeding. **A** qPCR analysis of hepatic mRNA expression of genes involved in lipogenesis, fatty acid oxidation, lipid secretion and lipid uptake (*n* = 5/group). **B**, **C** Western blot (**B**) and quantification (**C**) analyses of hepatic SREBP1 and FASN. **D**, **E** Western blot (**D**) and quantification (**E**) analyses of hepatic PPARα and CPT1A. **F** qPCR analysis of hepatic mRNA expression of PPARα target genes (*n* = 5/group). **G** Serum β-hydroxybutyrate measurements (*n* = 6/group). **H** Representative electron microscopy images (×2000 magnification, left panels; ×7000 magnification, middle and right panels) of hepatocytes from Gao-Binge ethanol-treated LoxP and LKO mice. **I**, **J** Numbers of total (**I**) and damaged (**J**) mitochondria were quantified in hepatocytes (*n* = 9/group) from Gao-Binge ethanol-treated LoxP and LKO mice. Data are presented as mean ± SD. *p < 0.05. In (**A**–**G**), *p < 0.05 vs PF LoxP; ^#^*p* < 0.05 vs AF LoxP. Scale bars, 5 µm.

To delineate the molecular mechanisms of Gao-Binge ethanol feeding-induced hepatic steatosis in Depdc5-LKO mice, we assessed the mRNA expression of key genes involved in the main pathways in hepatic lipid metabolism. Surprisingly, Gao-Binge ethanol feeding downregulated the mRNA expression of key regulators of de novo lipogenesis (DNL), including *Srebp1c*, *Fasn*, and *Acaca* in the livers of mice compared to pair-fed control mice, and *Depdc5* ablation further reduced their expression (Fig. 6A–C). These findings suggest that DNL is unlikely to contribute to the hepatic lipid accumulation observed in ethanol-fed Depdc5-LKO mice. In addition, hepatic mRNA levels of genes involved in lipid uptake (*Fatp2*, *Fatp5*) and very-low-density lipoprotein (VLDL) secretion (*Mttp*, *Apob*) were not significantly altered in pair- and ethanol-fed LoxP and Depdc5-LKO mice, indicating these two processes may not contribute to the development of alcohol-induced fatty liver in Depc5-LKO mice (Fig. 6A). Interestingly, the transcription of key genes for fatty acid oxidation, such as *Ppara*, *Cpt1a*, *Acox1*, was reduced after ethanol exposure and *Depdc5* deletion greatly exacerbated the reduction of their expression (Fig. 6A, D, E). As shown in Fig. 6F, the mRNA levels of PPARα downstream target genes such as *Acadvl*, *Acadm*, *Acadl*, *Cyp4a10*, *Cyp4a14*, and *Cyp4a32* were significantly decreased in the livers of pair- and ethanol-fed Depdc5-LKO mice. Consistent with the hypothesis that hepatic *Depdc5* deletion leads to impaired fatty acid oxidation, serum levels of β-hydroxybutyrate, a type of ketone body, were lower in Depdc5-LKO mice than in LoxP mice (Fig. 6G).

RNA-seq was used to determine alterations in the transcriptional profiles of liver tissues from alcohol-treated LoxP and Depdc5-LKO mice. As shown in Fig. S6A, volcano plot results showed the overall differentially expressed genes (DEGs) of the livers between ethanol-fed LoxP and LKO mice. RNA-seq analysis revealed that 335 genes were upregulated and 236 genes were downregulated in Depdc5-LKO livers compared to LoxP livers. KEGG pathway analysis further revealed enrichment of DEGs in multiple pathways (Fig. S6B, C). Consistent with our qPCR data, PPAR signaling pathway was one of the most enriched pathways associated with downregulated DEGs (Fig. S6C).

Mitochondrial dysfunction plays a dominant role in the pathogenesis of ALD [22]. By analyzing TEM images, we found obvious mitochondrial damage in hepatocytes of ethanol-fed Depdc5-LKO mice (Fig. 6H). While total numbers of hepatocyte mitochondria were only slightly reduced in Depdc5-LKO mice, numbers of damaged mitochondria in hepatocytes were markedly increased in Depdc5-LKO mice compared to LoxP mice (Fig. 6I, J).

### Fenofibrate administration rescues ethanol-induced liver pathologies in Depdc5-LKO mice

**Fig. 7:**
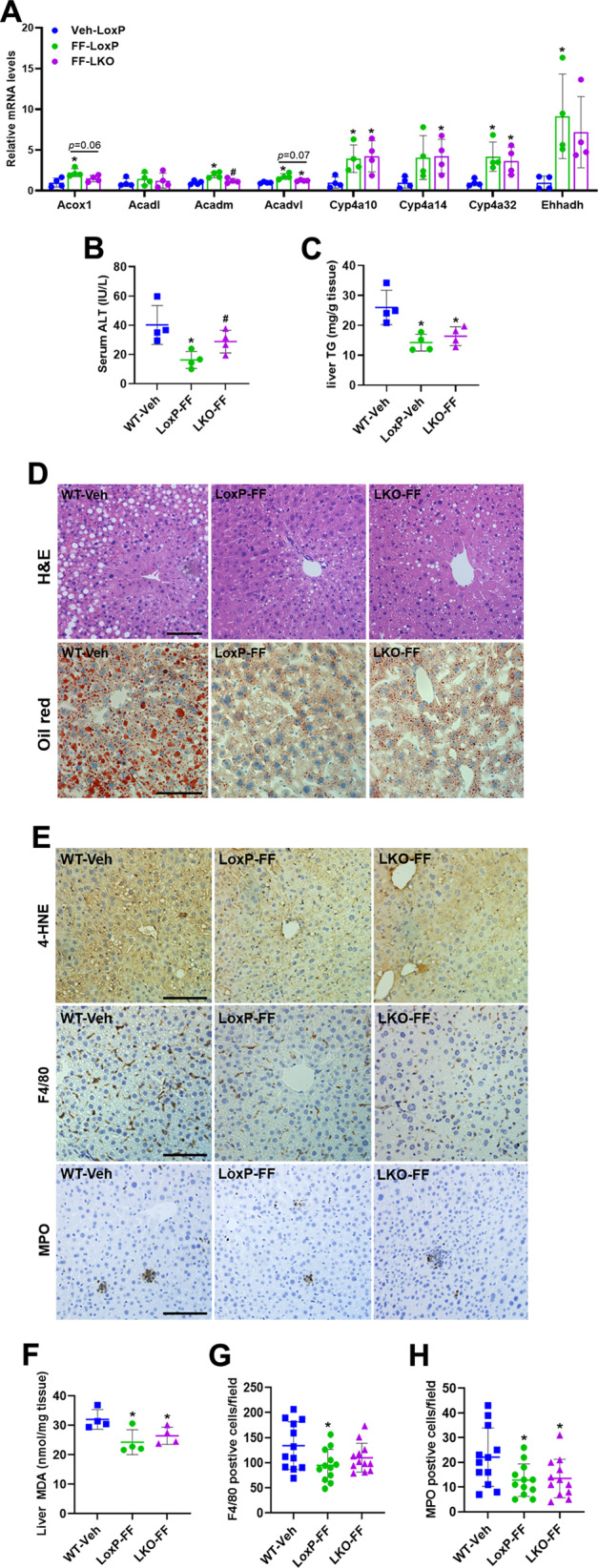
Fenofibrate treatment ameliorates ethanol-induced hepatic steatosis and inflammation in Depdc5-LKO mice. Three-month-old male WT, LoxP and Depdc5-LKO mice were subjected to Gao-Binge ethanol feeding. **A** qPCR analysis of hepatic mRNA expression of fatty acid oxidation-related genes (*n* = 4/group). **B**, **C** Serum ALT (**B**) and liver TG (**C**) measurements (*n* = 4/group). **D** Representative H&E (upper) and Oil Red O (lower) stained liver sections (200×). **E** IHC analysis of 4-HNE, F4/80, and MPO in liver sections (200×, *n* = 4/group). **F** Hepatic MDA measurements (*n* = 4/group). **G**, **H** Quantification of F4/80-positive cells (**G**) and MPO-positive cells (**H**) shown in (**E**). Data are presented as mean ± SD. *p < 0.05 vs WT-Veh; ^#^*p* < 0.05 vs LoxP-FF. Scale bars, 100 µm.

To determine whether impaired PPARα activity contributes to the ethanol-induced liver pathologies observed in Depdc5-LKO mice, we then treated ethanol-fed LoxP and Depdc5-LKO mice with fenofibrate, a clinically used PPARα agonist [23]. As expected, fenofibrate treatment stimulated the expression of PPARα target genes in the livers of both LoxP and Depdc5-LKO mice compared to vehicle-treated, ethanol-fed WT control mice (Fig. 7A). Gao-Binge ethanol feeding-induced liver injury and steatosis were largely normalized in both LoxP and Depdc5-LKO mice by fenofibrate administration (Fig. 7B–D). 4-HNE staining and hepatic MDA analysis also showed significant reduction of oxidative stress in both LoxP and Depdc5-LKO mice after fenofibrate treatment (Fig. 7E, F). IHC staining of F4/80 and MPO revealed that fenofibrate administration markedly reduced the number of macrophages and neutrophils in the liver tissues of both LoxP and Depdc5-LKO mice fed an ethanol diet (Fig. 7E, G, H).

## Discussion

Despite some progress has been made to elucidate the underlying mechanisms of ALD, the pathogenesis of ethanol-induced hepatic steatosis remains elusive. While some reports have indicated that ethanol exposure activates SREBP1c and the lipogenic pathway [24, 25], other studies have shown that ethanol exposure downregulates the expression of *Srebp1c* and lipogenesis-related genes [26, 27]. Furthermore, our current data also revealed that Gao-Binge ethanol feeding reduced hepatic expression of *Srepb1c* and other lipogenic genes in mice. One explanation for the suppression of lipogenic process by ethanol exposure is that ethanol and/or its metabolites impair insulin release and cause insulin resistance, and further inhibit the AKT signaling which is required for the activation of SREPB1c and the lipogenic pathway in the liver [28]. Therefore, the lipogenic pathway may not be a major contributor to the development of ethanol-induced fatty liver. Increased NADH/NAD^+^ ratio caused by ethanol oxidation in hepatocytes favors inhibition of fatty acid β-oxidation in the liver [28]. In addition, ethanol exposure stimulates adipose tissue lipolysis to generate lots of free fatty acids [29]. Therefore, the imbalance of excessive influx of fatty acids mobilized from adipocytes and the suppressed fatty acid oxidation capacity induce excessive fat accumulation in the liver, which is a key mechanism leading to ethanol-induced liver steatosis.

It is well known that mTORC1 activation upregulates SREBP1c expression and promotes lipid synthesis [30, 31]. However, studies of genetically engineered mice with liver-specific knockout of *Tsc1* have demonstrated that activation of mTORC1 in hepatocytes surprisingly protects against diet-induced hepatic steatosis [13, 14]. A recent study and our current data also revealed that mTORC1 hyperactivation caused by *Depdc5* deletion in the liver results in decreased lipid accumulation in mice fed a HFD [15]. Thus, all these results suggest that mTORC1 activation is not sufficient to stimulate SREBP1c and the DNL, and mTORC1-depedent feedback inhibition of AKT signaling underlies the defect in SREBP1c induction. Unexpectedly, we observed an opposite phenotype of lipid metabolism in ethanol-fed Depdc5-LKO mice, which hepatic *Depdc5* ablation leads to more severe hepatic steatosis and inflammation in mice exposed to ethanol. Since severe hepatic steatosis develops in Depdc5-LKO mice administrated with ethanol by two ethanol feeding protocols (Gao-Binge model and LD 5W model), we believe that hepatic mTORC1 activation indeed aggravates ethanol-induced steatosis. However, the question is why sustained activation of mTORC1 in hepatocytes protects liver from diet-induced steatosis but exacerbates hepatic steatosis induced by ethanol exposure? The divergent outcomes are likely attributed to the differences in the pathogenesis between these two fatty liver models. Recent evidence and our findings have shown that sustained activation of mTORC1 does not affect the lipid uptake and VLDL secretion pathways, but notably suppresses the expression of genes involved in both DNL and fatty acid oxidation in the livers of mice treated with either HFD or ethanol [13, 14]. As we know, diet-induced excess lipid accumulation in the liver is largely through the enhancement of DNL pathway which is primarily controlled by SREBP1c [32]. Therefore, suppressed lipogenesis in hepatocytes caused by sustained mTORC1 activation is sufficient to protect the liver from HFD-induced steatosis. However, in the progression of alcoholic liver steatosis, the relative contribution of DNL to alcoholic liver steatosis is less important because ethanol also stimulates adipose tissue lipolysis to release plenty of free fatty acids. Suppressed lipogenesis mediated by mTORC1 hyperactivation is insufficient to prevent excess accumulation of lipids in the liver induced by ethanol exposure. Conversely, mTORC1 activation further inhibits the expression of PPARα and its target genes, and exacerbates the ethanol-induced fatty acid oxidation defects, thereby leading to alcoholic liver steatosis.

As we reported, sustained mTORC1 activation in hepatocytes suppresses the expression of PPARα and other β-oxidation-related genes. However, the mechanisms by which mTORC1 hyperactivation inhibits PPARα expression and activity are still not very clear. Sengupta et al. have demonstrated that mTORC1 regulates PPARα and ketogenesis via controlling the subcellular localization of NcoR1, co-repressor of PPARα [33]. Moreover, Kim et al. have revealed that S6K2, a downstream effector of mTORC1, directly interacts with NcoR1 and recruits it to the nucleus to suppress PPARα activity [34]. But it remains unclear whether S6K2 recruits NcoR1 to suppress PPARα activity in ethanol-treated hepatocytes.

Accumulating evidence has indicated that persistent suppression of mTORC1 signaling by *Raptor* knockout in the liver or by chronic rapamycin treatment inhibits lipid accumulation but induces inflammation and hepatocellular damage [20]. Consistently, we have also observed that adenovirus-mediated shRNA knockdown of *Raptor* in control mice rescued the alcoholic liver steatosis, but even exacerbated liver injury and inflammation. However, pharmacological inhibition of mTORC1 signaling by Torin 1 treatment (20 mg/kg per day for 5 days) markedly reverses almost all of the ethanol-induced liver abnormalities we observed in Depdc5-LKO mice, including liver steatosis, injury, and inflammation. The phenotypic discrepancy may attribute to the different extent of mTORC1 inhibition by these two approaches. Adenoviral shRNA expression system stably induces specific *Raptor* gene silencing in hepatocytes, which results in a persistent mTORC1 suppression. Since the in vivo half-life of Torin 1 is much shorter than that of rapamycin [21], it is reasonable to speculate that Torin 1 (20 mg/kg/day) might inhibit mTORC1 signaling more modestly than shRNA knockdown or even rapamycin, thereby maintaining the hepatic mTORC1 activity in a proper level to avoid liver injury and inflammation caused by persistent mTORC1 suppression. It is worth noting, Torin 1 is an ATP-competitive mTOR inhibitor that directly inhibits both mTORC1 and mTORC2 complexes [35]. Therefore, the differential effects of Ad-shRaptor knockdown and Torin 1 treatment may also be explained by the different inhibitory mechanisms of these two approaches. The exact role mTORC2 signaling plays in ethanol-induced liver steatosis and injury needs to be further studied.

**Fig. 8:**
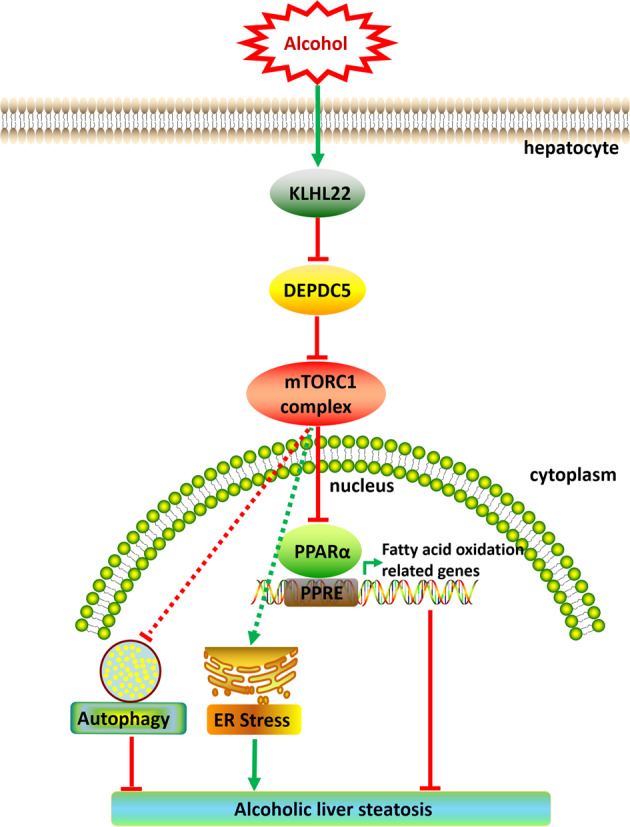
A proposed model for the dysregulation DEPDC5-mTORC1 axis in the pathogenesis of alcohol-induced hepatic steatosis. Chronic ethanol consumption leads to KLHL22-mediated degradation of DEPDC5, then activates mTORC1 signaling. Aberrant activation of mTORC1 reduces the expression of PPARα and its target genes, thereby resulting in alcohol-induced liver steatosis.

In summary, we demonstrate that hepatocyte-specific *Depdc5* deletion hyperactivates mTORC1 signaling and exacerbates ethanol-induced liver steatosis and inflammation. Repressed PPARα-driven fatty acid oxidation caused by sustained mTORC1 activation contributes to the development of ethanol-induced hepatic steatosis and inflammation. Pharmacological inhibition of mTORC1 signaling by Torin 1 or activation of PPARα by fenofibrate rescues the ethanol-induced liver abnormalities caused by mTORC1 hyperactivation. Our data provide the first in vivo evidence illustrating the hepatocyte-intrinsic roles of hyperactivation of mTORC1 signaling in regulating the pathogenesis of ALD (Fig. 8).

## Supplementary information

Supplementary materials.

Uncropped images of blots.
