# Supplementary material for: Depdc5 deficiency exacerbates alcohol-induced hepatic steatosis via suppression of PPARα pathway
Source: Cell Death Dis. 2021 Jul 15;12(7):710. doi: 10.1038/s41419-021-03980-6 (PMC8282792; doi:10.1038/s41419-021-03980-6)
Supplement: Supplementary file 1 — Supplementary materials. [file 41419_2021_3980_MOESM1_ESM.docx]

**Supplementary figure legends**

**Figure S1. Depdc5-LKO mice are protected from diet-induced hepatic steatosis.** 6-week-old male LoxP and Depdc5-LKO mice were treated with high-fat diet (60% calories from fat) for 4 months. (A-C) Body weights (A), liver weights (B) and liver TG levels (C) of LoxP and LKO mice (n=6/group). (D) Representative H&E images of liver sections. Data are presented as mean ± SD. *p<0.05. Scale bars, 100µm.

**Figure S2. Hepatic *Depdc5* deletion impairs autophagy and aggravates ethanol-induced ER stress.** 2-3-month-old LoxP and Depdc5-LKO mice were subjected to Gao-Binge ethanol feeding. (A, B) Liver lysates were subjected to western blot analysis to examine autophagy markers (A) and ER stress markers (B).

**Figure S3. Hepatic loss of *Depdc5* exacerbates liver steatosis and injury in mice treated with LD 5W ethanol feeding.** 2-3-month-old female LoxP and Depdc5-LKO mice were subjected to LD 5W ethanol feeding. (A-C) Liver weight to body weight ratios (A), serum ALT levels (B) and liver TG levels (C) were determined (n=4-5/group). (D) Representative H&E images of liver sections. Data are presented as mean ± SD. *p<0.05 vs WT-PF; #p<0.05 vs LoxP-AF. Scale bars, 100µm.

**Figure S4. Hepatic *Depdc5* ablation does not exacerbate liver fibrosis in mice after Gao-Binge ethanol feeding.** 2-3-month-old LoxP and Depdc5-LKO mice were subjected to Gao-Binge ethanol feeding. (A) Western blot analysis of hepatic αSMA. (B) qPCR analysis of hepatic mRNA expression of fibrogenic genes (n=5/group). (C) Representative images of Sirius red staining in liver sections. Data are presented as mean ± SD. Scale bars, 100µm.

**Figure S5. Adenovirus-mediated hepatic *Raptor* knockdown reverses liver steatosis but worsens liver injury and inflammation in control mice treated with Gao-Binge ethanol feeding.** 3-month-old male LoxP and LKO mice were subjected to Gao-Binge ethanol feeding. (A) Liver lysates were subjected to western blot analysis to examine RAPTOR, mTORC1 signaling and AKT phosphorylation. (B-D) Liver weight to body weight ratios (B), serum ALT levels (C) and liver TG levels (D) were determined (n=5/group). (E) Representative H&E images of liver sections. (F) Hepatic macrophages and neutrophils were examined by immunohistochemistry staining with anti-F4/80 and anti-MPO antibodies (n=4/group). (G, H) Quantification of F4/80 positive cells (G) and MPO positive cells (H) in Panel F. Data are presented as mean ± SD. *p<0.05 vs shGFP LoxP; #p<0.05 vs shRaptor LoxP. Scale bars, 100µm.

**Figure S6. RNA-seq analysis of liver genes in the alcohol-fed LoxP and Depdc5-LKO mice.** 3-month-old male LoxP and LKO mice were subjected to Gao-Binge ethanol feeding. (A) Volcano graph showing up-regulated and down-regulated genes in the livers of LKO mice compared to LoxP mice (n=3/group). (B and C) KEGG annotation for the upregulated (B) and downregulated (C) genes expressed in the livers between LKO and LoxP mice (n=3/group).

**Fig. S1**


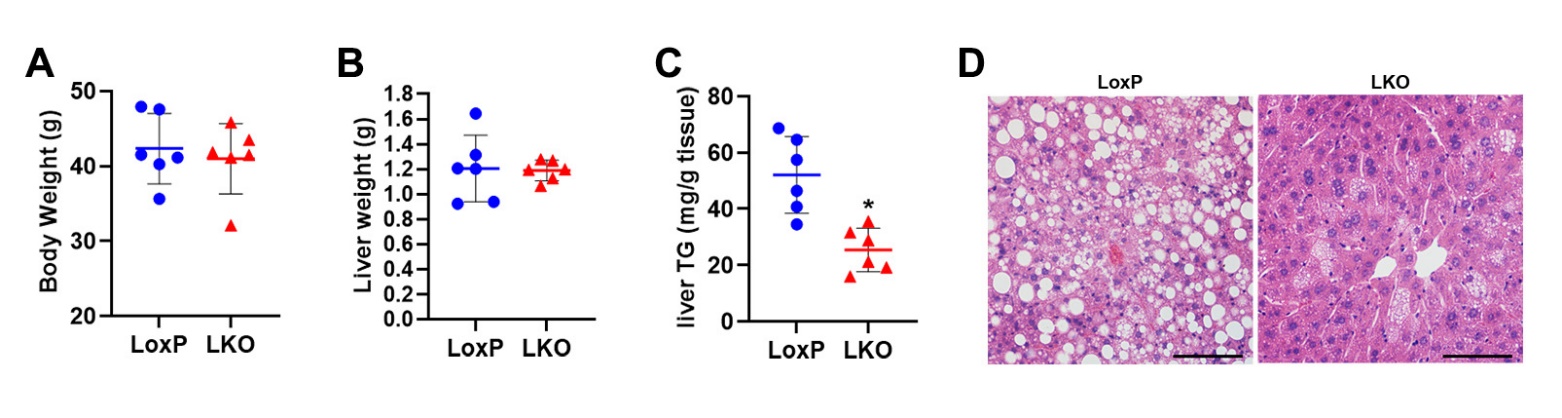


**Fig. S2**


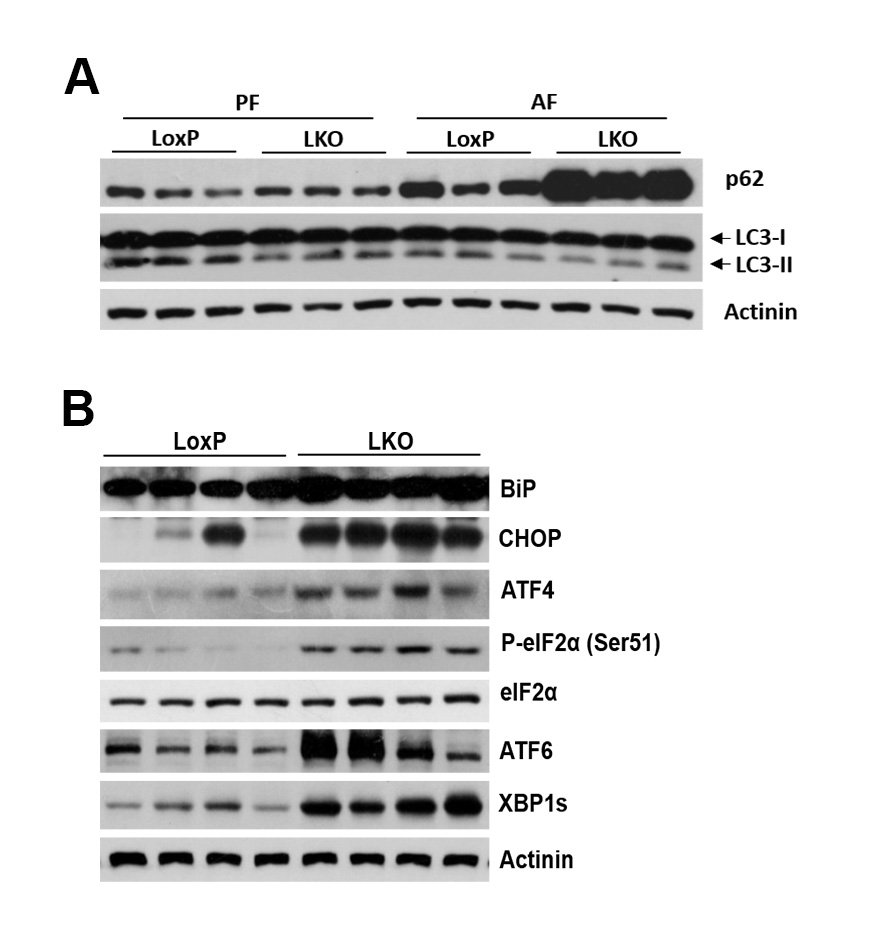


**Fig. S3**


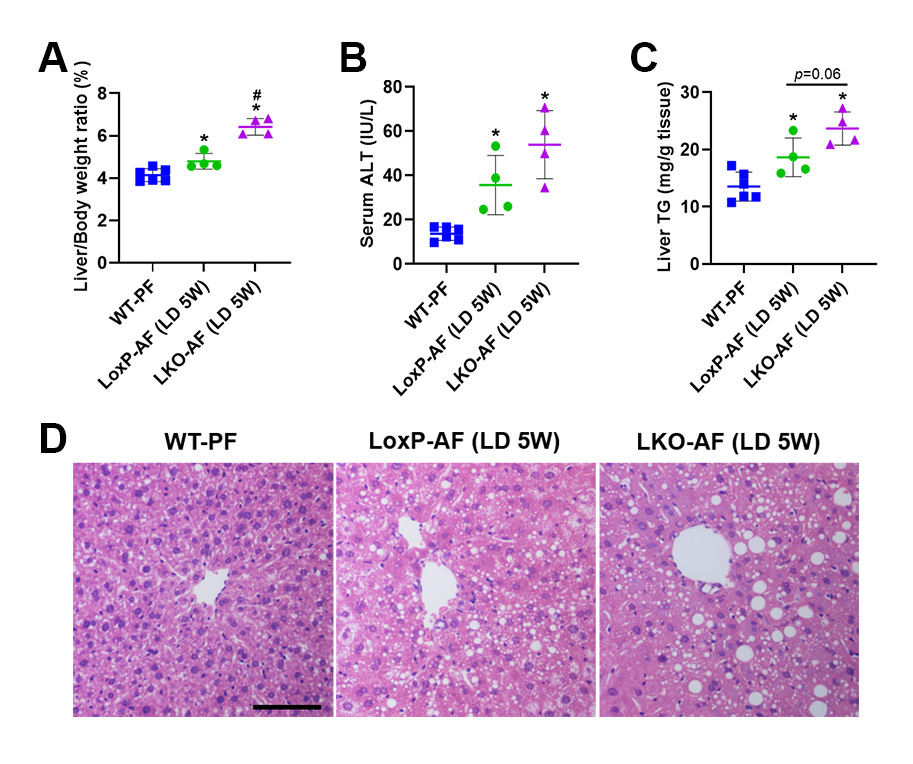


**Fig. S4**


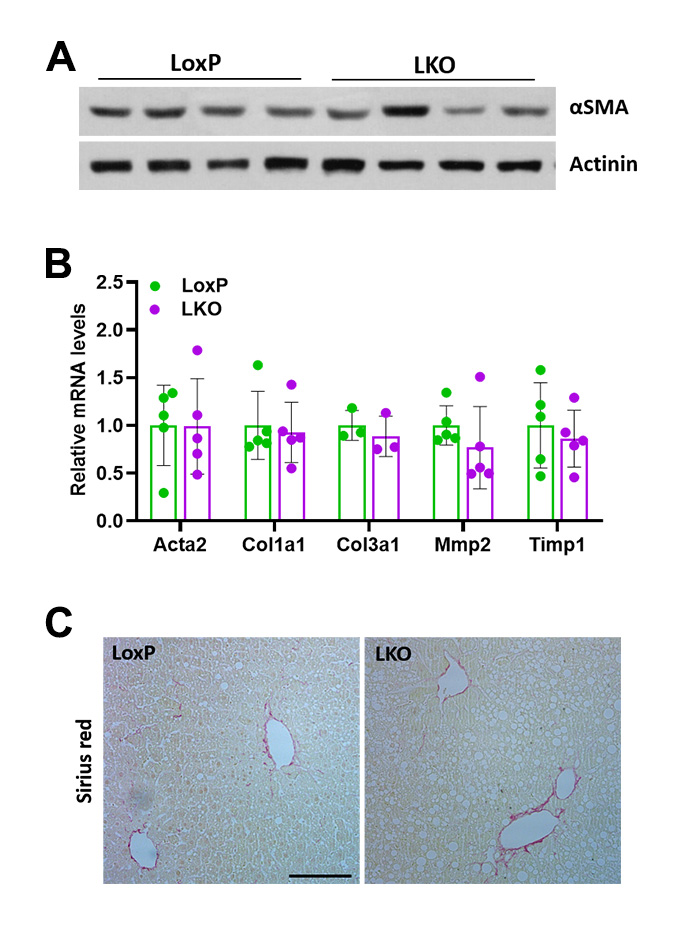


**Fig. S5**


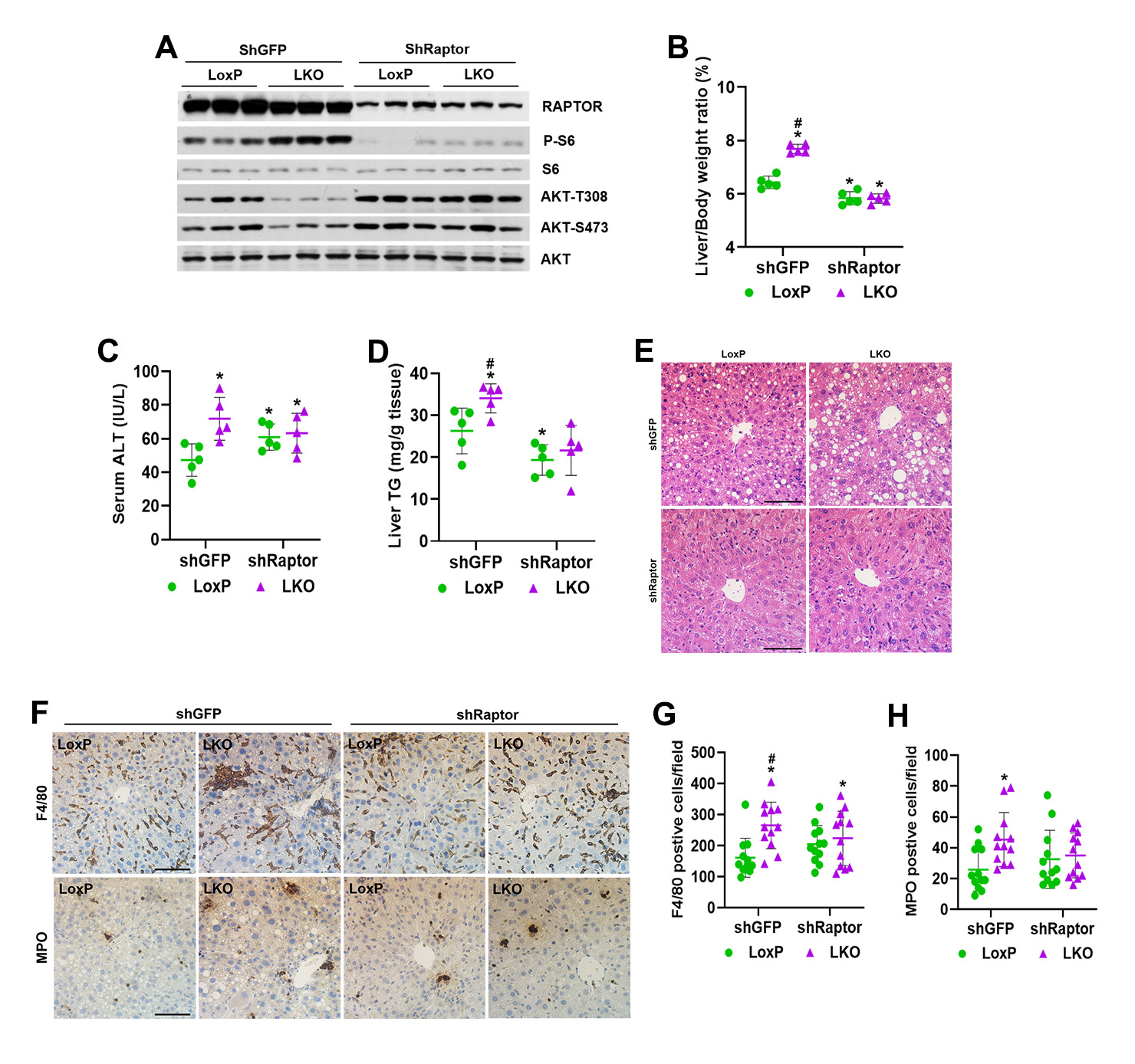


**Fig. S6**


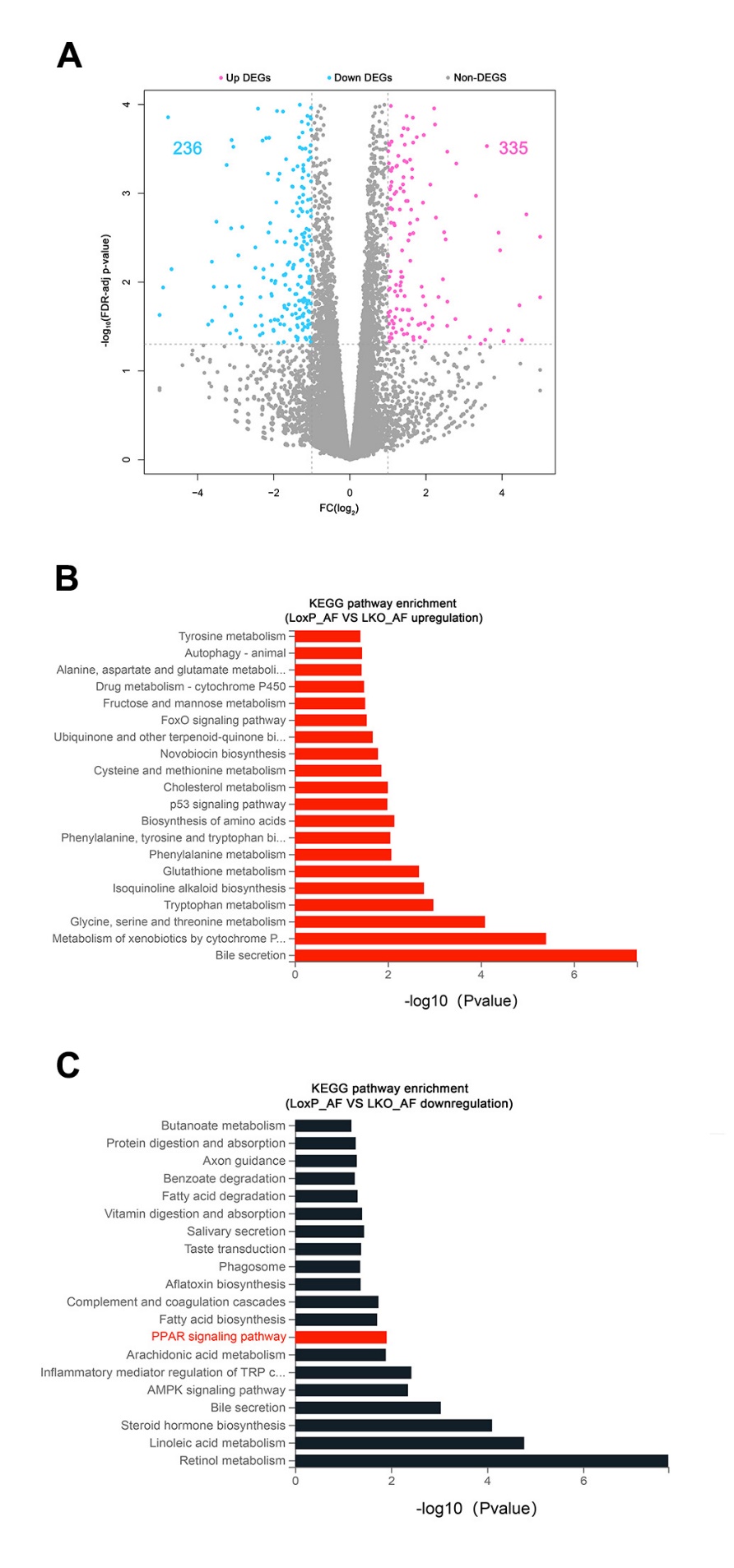


**Table S1. Characteristics of the AH patients**

| AH | Age |  |  | PT | WBC | HB | PLT | AST | ALT | ALP | ALB | TBIL | MELD | DCM |
| --- | --- | --- | --- | --- | --- | --- | --- | --- | --- | --- | --- | --- | --- | --- |
| Pts ID | (yr) | Sex | Race | (sec) | (x10^3^/ul) | (g/dL) | (x10^3^/ul) | (U/L) | (U/L) | (U/L) | (g/dL) | (mg/dL) |  |  |
| AH1 | 32 | M | White | 18.4 | 23.65 | 8 | 147 | 141 | 76 | 188 | 3.2 | 38.8 | 34 | Yes |
| AH2 | 34 | F | White | 17 | 5.7 | 7.4 | 119 | 144 | 49 | 87 | 3.7 | 27.7 | 34 | Yes |
| AH3 | 49 | F | White | 16.8 | 16.98 | 7.6 | 27 | 127 | 52 | 175 | 2.9 | 14.2 | 38 | Yes |
| AH4 | 48 | M | White | 41 | 8.65 | 8.1 | 66 | 121 | 41 | 110 | 4 | 48 | 45 | Yes |
| AH5 | 41 | M | White | 34.7 | 14.02 | 7.2 | 57 | 95 | 44 | 129 | 2.9 | 33.4 | 34 | Yes |
| AH6 | 61 | M | White | 23.6 | 7.86 | 9.3 | 45 | 71 | 36 | 98 | 3.4 | 35.6 | 38 | Yes |

AH, alcoholic hepatitis; Pts, patients; PT, prothrombin time; WBC, white blood cells; HB, hemoglobin; PLT, platelet; AST, aspartate transaminase; ALT, alanine transaminase; ALP, alkaline phosphatase; ALB, albumin; TBIL, total bilirubin; MELD, model for end-stage liver disease score; DCM, decompensation.

**Table S2. The information of the primary antibodies used in this study**

| Antibody | Company | Cat# | Species | Dilution |
| --- | --- | --- | --- | --- |
| DEPDC5 | Abcam | Ab185565 | rabbit | 1:500 |
| P-S6 | Cell signaling technology | 4858 | rabbit | 1:4000 |
| RPS6 | Proteintech | 14823-1-AP | rabbit | 1:1000 |
| P-P70S6K | Cell signaling technology | 9234 | rabbit | 1:1000 |
| P70S6K | Cell signaling technology | 2708 | rabbit | 1:500 |
| AKT-S473 | Cell signaling technology | 4060 | rabbit | 1:3000 |
| AKT-T308 | Cell signaling technology | 13038 | rabbit | 1:1000 |
| AKT | Proteintech | 10176-2-AP | rabbit | 1:1000 |
| KLHL22 | Proteintech | 16214-1-AP | rabbit | 1:1000 |
| Actinin | Proteintech | 11313-2-AP | rabbit | 1:5000 |
| GAPDH | Proteintech | 60004-1-Ig | mouse | 1:10000 |
| P62 | Proteintech | 18420-1-AP | rabbit | 1:2000 |
| LC3B | ABclonal | A19665 | rabbit | 1:2000 |
| SREBP1 | Santa Cruz | sc-13551 | mouse | 1:400 |
| FASN | Proteintech | 10624-2-AP | rabbit | 1:5000 |
| PPARɑ | Proteintech | 15540-1-AP | rabbit | 1:3000 |
| CPT1A | Proteintech | 15184-1-AP | rabbit | 1:4000 |
| BIP | Proteintech | 11587-1-AP | rabbit | 1:3000 |
| CHOP | Proteintech | 15204-1-AP | rabbit | 1:1000 |
| ATF4 | Cell signaling technology | 11815 | rabbit | 1:2000 |
| P-elF2ɑ | Cell signaling technology | 3398 | rabbit | 1:1000 |
| ATF6 | Cell signaling technology | 65880 | rabbit | 1:1000 |
| XBP1 | Abcam | Ab220783 | rabbit | 1:1000 |
| ɑSMA | Abcam | Ab124964 | rabbit | 1:40000 |
| RAPTOR | Proteintech | 20984-1-AP | rabbit | 1:1000 |
| MPO | Abcam | ab208670 | rabbit | 1:200 |
| F4/80 | Cell signaling technology | 70076 | rabbit | 1:400 |

**Table S3. The sequence information of the primers used for reverse transcriptional quantitative PCR**

| Genes | Species | Forward | Reverse |
| --- | --- | --- | --- |
| Depdc5 KO | mouse | GCAAGTCAAGTCGCTTAAGGA | GGTCTTAGCCGGAATACTTGAG |
| Klhl22 | mouse | CCAAGGATTTCGAGCTGAGTC | CCTGCCACAGCATAGATGTAC |
| Ccl2 | mouse | CCCAATGAGTAGGCTGGAGA | TCTGGACCCATTCCTTCTTG |
| Il1b | mouse | TGTGAAATGCCACCTTTTGA | GGTCAAAGGTTTGGAAGCAG |
| Il6 | mouse | CAAAGCCAGAGTCCTTCAGAG | GAGCATTGGAAATTGGGGTA |
| Tnf | mouse | GGCCTCCCTCTCATCAGTTC | CACTTGGTGGTTTGCTACGA |
| Srebf1 | mouse | TGCATTTTCTGACACGCTTC | GAGGCACTGAGCCACTCTG |
| Fasn | mouse | CTGCCACAACTCTGAGGACA | TTCGTACCTCCTTGGCAAAC |
| Acaca | mouse | GGCCAGTGCTATGCTGAGAT | TATCACACAGCCAGGGTCAA |
| Ppara | mouse | CTGCAGAGCAACCATCCAGAT | GCCGAAGGTCCACCATTTT |
| Cpt1a | mouse | GGAGAGAATTTCATCCACTTCCA | CTTCCCAAAGCGGTGTGAGT |
| Acox1 | mouse | TACGTGCAGCCAGATTGGTA | AAGTCAAGTTCCACGCCACT |
| Mttp | mouse | ATGATCCTCTTGGCAGTGCTT | TGAGAGGCCAGTTGTGTGAC |
| Apob | mouse | TGAATGCACGGGCAATGA | GGCATTACTTGTTCCATGGTTCT |
| Slc27a2 | mouse | TATGACGTGGAGAAGGACGAG | GCCAATAAATGGTGTGAGCTG |
| Slc27a5 | mouse | CATGGCGTAACAGTGATCTTG | GCGCACTGTATGTATCTTGTC |
| Acadvl | mouse | GCAGGCACCATGAAATCCCTC | AGAATAGCCATCCGAGCCAGC |
| Acadm | mouse | CAGGTTTCAAGATCGCAATGG | ATACTTCGTGGCTTCGTCTAG |
| Acadl | mouse | GATTGCCAGCTAATGCCTTAC | GCCAACTCAGCAATTAAGAGC |
| Cyp4a10 | mouse | GAACTTCCCAAGTGCCTTTCC | CCTTTGGATCTGATCGCCCC |
| Cyp4a14 | mouse | TCGGGGAGCAATATACGAGTCC | GGAGCAAACCATAACCAATCCAG |
| Cyp4a32 | mouse | GCAGAGAGGAAGTTCAGAGC | ACAGGTGGGTAGAGCCTTAG |
| Hadhb | mouse | CACTGCGTTCTCATAGTCTGG | ATAGGGTTTGATGAACGCAGG |
| Ehhadh | mouse | GGGCTGTCACTATCGGATTGC | GCAACAGGAACTCCAACGACC |
| Acata2 | mouse | AGGCACCACTGAACCCTAAG | GACAGCACAGCCTGAATAGC |
| Col1a1 | mouse | CACCTGGTCCACAAGGTTTC | CCCATCATCTCCATTCTTGC |
| Col3a1 | mouse | TGCTGGAAAGGATGGAGAGT | TGGGCCTTTGATACCTGGAG |
| Mmp2 | mouse | ACTCCGGAGATCTGCAAACA | ACTGTCCGCCAAATAAACCG |
| Timp1 | mouse | CATGGAAAGCCTCTGTGGAT | CTCAGAGTACGCCAGGGAAC |
