# Supplementary material for: Depdc5 deficiency exacerbates alcohol-induced hepatic steatosis via suppression of PPARα pathway
Source: Cell Death Dis. 2021 Jul 15;12(7):710. doi: 10.1038/s41419-021-03980-6 (PMC8282792; doi:10.1038/s41419-021-03980-6)

Uncropped images of blots for figure 1C


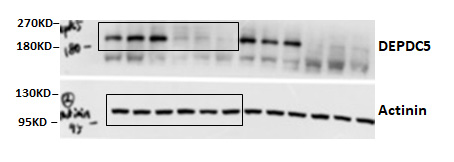


Uncropped images of blots for figure 1J


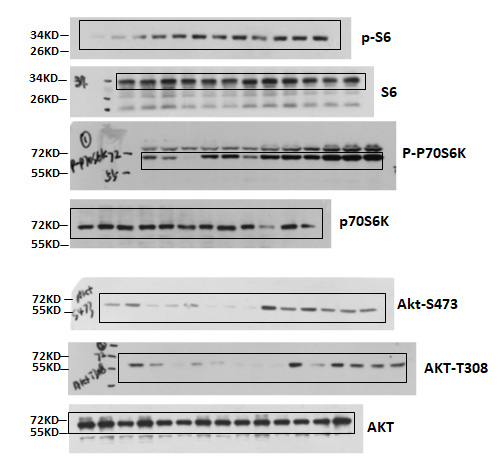


Uncropped images of blots for figure 2A


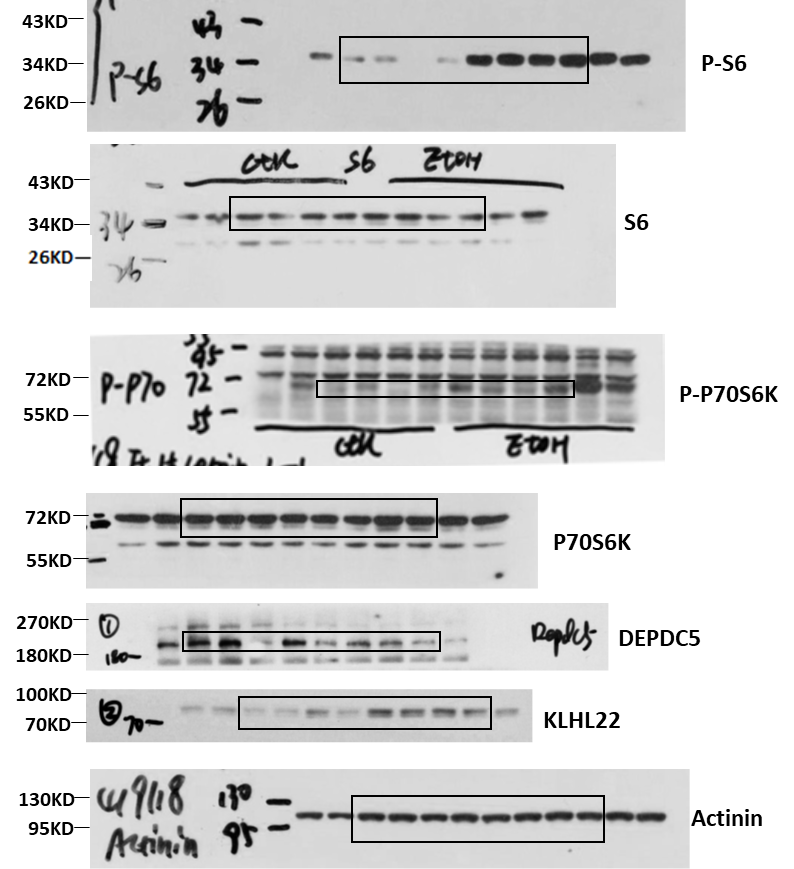


Uncropped images of blots for figure 2C


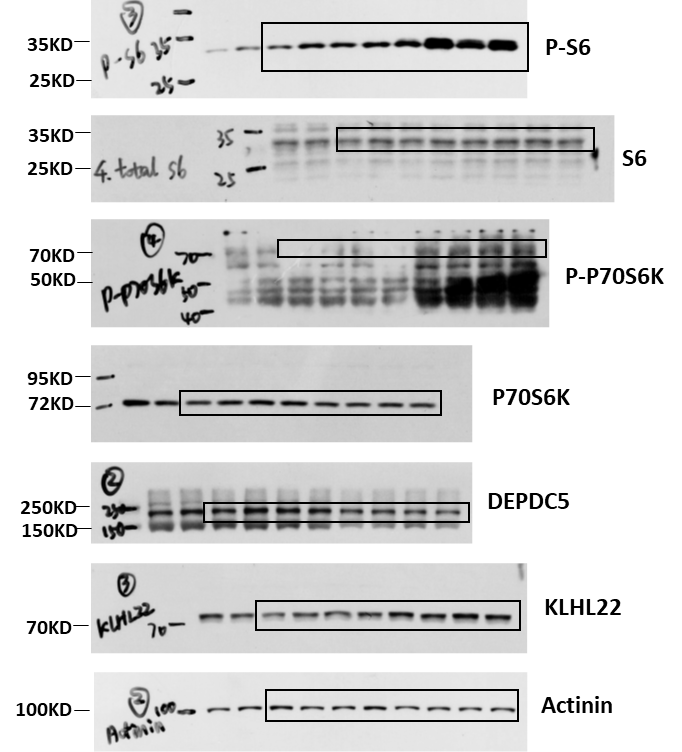


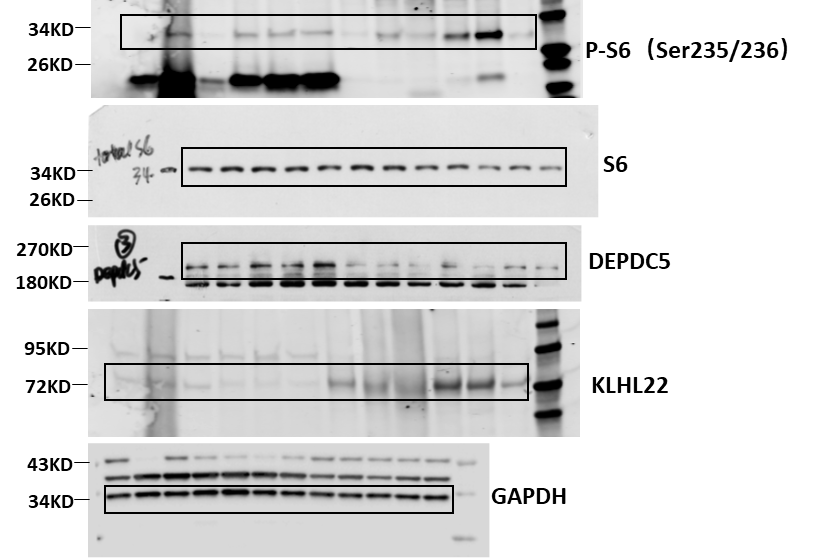
Uncropped images of blots for figure 2G

Uncropped images of blots for figure 3A


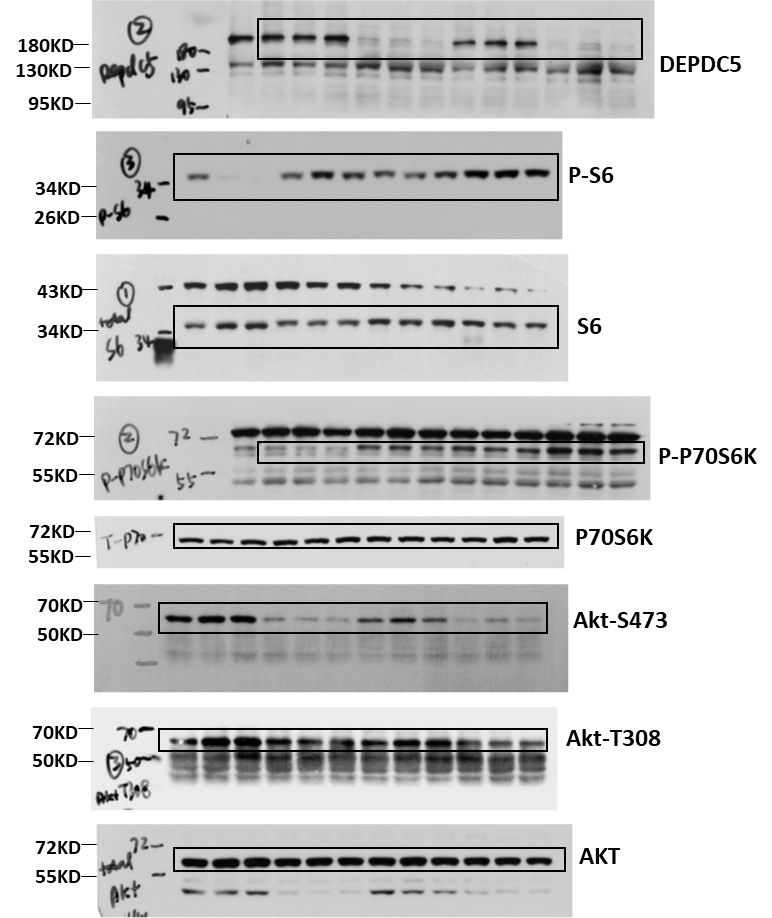


Uncropped images of blots for figure 5A


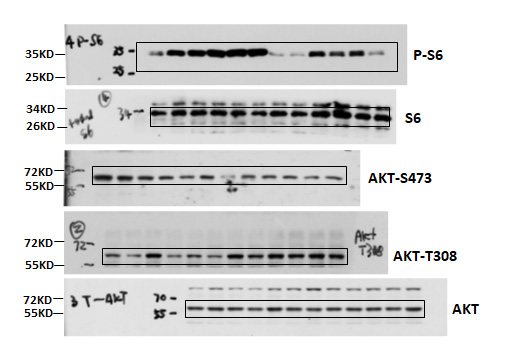


Uncropped images of blots for figure 6B


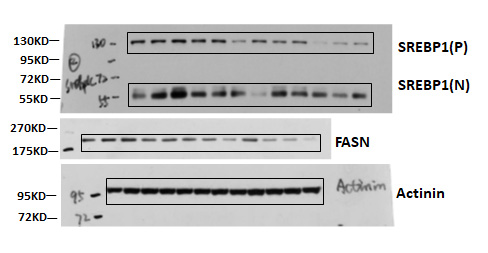


Uncropped images of blots for figure 6D


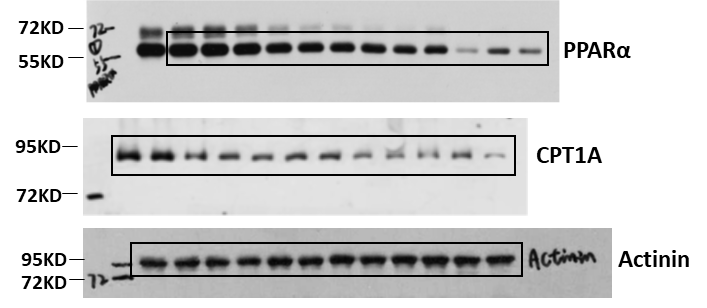


Uncropped images of blots for figure S2A


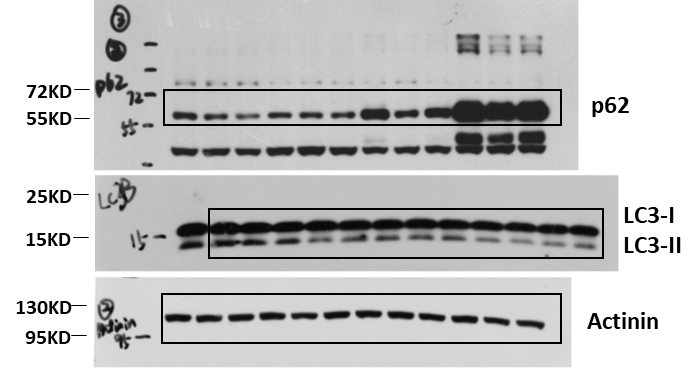


Uncropped images of blots for figure S2B


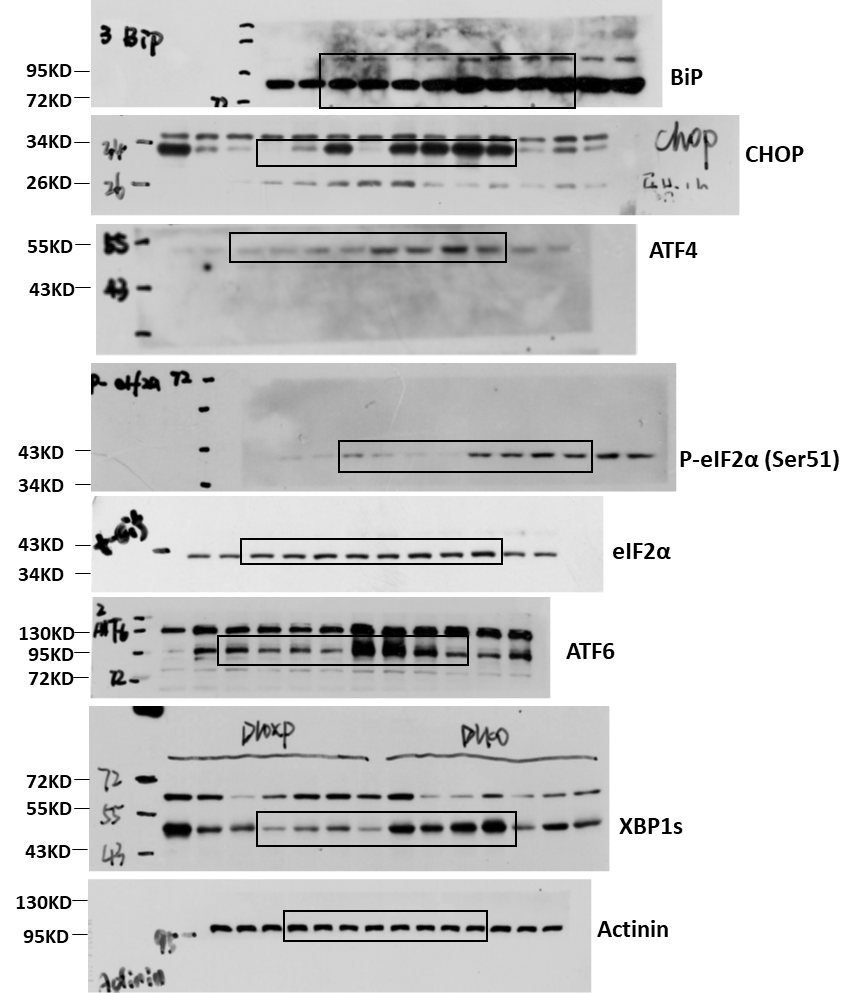


Uncropped images of blots for figure S4A


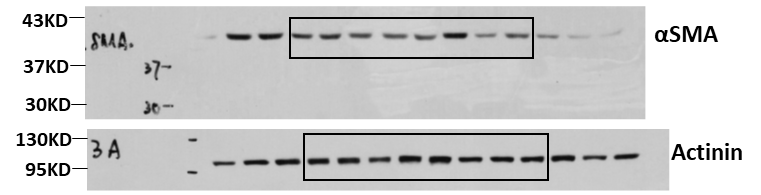


Uncropped images of blots for figure S5A


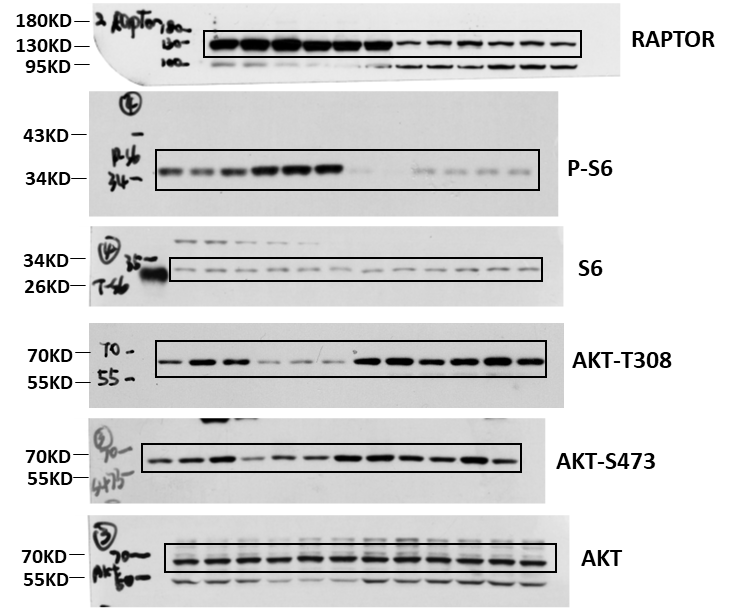

Supplement: Supplementary file 2 — Uncropped images of blots. [file 41419_2021_3980_MOESM2_ESM.docx]
